# Do Juveniles Who Have Committed Sexual Offenses Have Higher Callous-Unemotional Traits Compared to Juveniles Who Have Committed General Offenses? A Systematic Review

**DOI:** 10.3390/bs14070525

**Published:** 2024-06-24

**Authors:** Eduarda Ramião, Andreia Geraldo, Patrícia Figueiredo, Ricardo Barroso, Fernando Barbosa

**Affiliations:** 1 Laboratory of Neuropsychophysiology, Faculty of Psychology and Educational Sciences, University of Porto, R. Alfredo Allen, 4200-135 Porto, Portugal; p7688@ulusofona.pt (A.G.); patricia.figueiredo@ulusofona.pt (P.F.);; 2 Escola Superior de Saúde, Polytechnic Institute of Porto (ESS-IPP), 4200-072 Porto, Portugal; 3 HEI-Lab: Digital Human-Environment Interaction Labs, Lusófona University, 1749-024 Lisboa, Portugal; 4 Departamento de Educação e Psicologia, University of Trás-os-Montes and Alto Douro, 5000-801 Vila Real, Portugal; rbarroso@utad.pt; 5 Laboratory of Research of Human Sexuality (Sexlab), Faculty of Psychology and Education Sciences, University of Porto, 4099-002 Porto, Portugal; 6 U.North Psychology Consortium, Portugal

**Keywords:** juveniles who have committed offenses, sexual offenses, callous-unemotional traits

## Abstract

The importance of assessing psychopathic traits in juveniles who have committed sexual offenses has been established in individuals who demonstrate a particularly severe and violent pattern of behavior. Additionally, the assessment of these traits in other juveniles might be relevant considering that higher levels of these traits represent an increased probability of the juvenile committing offenses. This study is a systematic review of the literature about the presence of callous-unemotional (CU) traits in juveniles who have committed sexual and non-sexual offenses, in order to ascertain eventual differences between these groups regarding the presence of CU traits. Studies were obtained from multiple databases, with predefined exclusion and inclusion criteria, according to PRISMA-P guidelines. A total of 18 studies were reviewed and included in the final analysis. The 18 studies used measures of CU traits and reported descriptive categories of CU traits in juveniles who have committed general offenses or juveniles who have committed sexual offenses. Meta-analytic procedures such as pooled means, pooled variances, and pooled standard deviations are presented in this study. The main conclusion obtained is that juveniles who have committed general offenses present higher levels of CU traits compared to juveniles who have committed sexual offenses. Although the review highlights limitations in the literature, the identification of these characteristics in different types of juveniles who have committed offenses is important to shed light on the phenomenon and develop interventions better suited to their characteristics. Recommendations for future research are also presented.

## 1. Introduction

Juvenile delinquency has a polymorphous character that encompasses several forms of behaviors, including both non-violent/non-aggressive (e.g., truancy, substance use) and violent behaviors, in which others may be intentionally harmed. To understand the violent criminal behavior of juveniles should be a priority in the social sciences field, as well as to learn to better deal with youths that have committed these offenses and who are integrated in the juvenile justice system [1,2].

Violent behavior might include, among others, interpersonal aggressions. These types of aggression are usually divided into verbal (e.g., insulting, threatening, teasing), physical (e.g., fighting, attacking, assaulting) or sexual [3]. Sexual violence is a significant worldwide societal problem [4]. The sexual behavior characteristics that define the presence or absence of this type of violence are the nature of the relationship and the presence of consent, equality, and coercion [5]. In relation to atypical sexual behavior and criminal characteristics, three distinct groups of persons emerged, namely individuals with coercive sexual interests (e.g., rapists), individuals who committed child sex offenses, and individuals with sexual pedophile interests [6]. Despite this typological distinction of individuals who have committed sexual offenses, it is important to mention that it is not determinist [6]. On one hand, individuals may only commit a single crime during their lives. On the other hand, specialist offenders may commit various sexual crimes with recidivism. Another group, designated as versatile/generalist offenders, may commit several separate crimes [7,8,9], which may be of a sexual nature, related to theft or assault, or related to substance abuse.

There are several studies that present biopsychosocial characteristics as risk factors to commit aggression and delinquent and/or antisocial behavior [9,10,11]. These may include characteristics of the juvenile (e.g., neuropsychological deficits, personality traits) and characteristics of the social context (e.g., parental neglect, social problems), which may influence their development [10,12,13]. Calkins and Keane [14] refer that studying the factors that indicate the presence of more serious antisocial tendencies allows the possibility of refining knowledge about the types of aggression including sexual offenses, risk patterns, and consequent risk assessment. Personality traits are considered a critical factor for understanding the development of serious antisocial careers [10], specifically psychopathic characteristics, which include affective deficits (e.g., lack of guilt and empathy), interpersonal deficits (e.g., narcissism, use of others to obtain gain), and behavioral problems (e.g., impulsivity) [10,12]. Psychopathy has been linked to aggressive behavior [15] and to the manifestation of a reduced responsivity to affective stimuli in youths [16].

The importance of assessing these characteristics in juveniles who have committed either sexual or non-sexual offenses (JOs) has been well established in individuals that demonstrate a particularly severe and violent pattern in their offenses in community and forensic samples [17,18,19]. These individuals tend to exhibit high callous-unemotional (CU) traits, which is the counterpart of the affective component of adult psychopathy [12]. The CU traits play an important role to identify limited prosocial emotions in these individuals and may lead to a more severe and aggressive pattern of behavior problems [14]. For example, Knight and Guay [20] reported that CU traits are specifically important in predicting increased violence, namely in sexual offenses. These traits allow the characterization of a subset of juveniles with conduct problems, exhibiting a lack of remorse and empathy, uncaring behaviors, and an inability to express emotion [12,21,22] and are moderately stable from late childhood to early adolescence [2,12]. Additionally, JO with high CU traits present a particularly severe and stable pattern of conduct problems and delinquent behavior and tend to present more evidence of substance abuse and higher rates of aggression, including violent and sexual offenses [2,10,21,23]. Nevertheless, higher CU traits in juveniles, for example, have been related to more frequent contacts with the police, violent and property delinquency, and substance abuse, when compared with peers with low CU traits [24].

On the one hand, in a meta-analysis by Eden and colleagues [25], with a total of 2867 juveniles who have committed offenses across all studies, CU traits’ measures have been associated with general violence or violent recidivism. Similarly, Frick and Dickens [26] reported associations between CU characteristics and more severe conduct disorders, delinquency, and aggression from early childhood to present adulthood. Corroborating what was mentioned earlier, these studies provide evidence that CU traits are associated with a particularly severe pattern of antisocial behavior. On the other hand, a meta-analysis by Seto and Lalumiere [13] identified that juveniles who have committed sexual offenses (JSOs) did not differ from juveniles who have committed general offenses (JGOs), and both groups tended to have above-average scores relative to norms on standardized personality measures. However, it is necessary to note that most studies focused on CU characteristics and their association with antisocial behavior in general, with sexual aggression being only a component. Additionally, previous studies indicate that JSOs constitute a unique group with specific characteristics, distinct from other delinquents [13]. This meta-analysis found a significant difference in studies comparing the two types of juveniles who have committed offenses (for example, criminal histories, antisocial peers, substance use problems), but in relation to CU traits, there are no significant differences. It is important to note that these authors’ study, despite providing important insights, focuses essentially on the various characteristics of antisocial behavior. This highlights the importance of seeking more detailed evidence on the differences in CU traits between young sexual and non-sexual offenders, thus contributing to a more nuanced and accurate understanding of the characteristics of these individuals.

Taking into account the heterogeneity in juvenile delinquency [27] and in the existing literature, it is not possible so far to reach a robust conclusion on the characteristics of CU traits that differentiate the types of juveniles who have committed offenses. The main goal of this systematic review is to examine the available literature regarding the association between CU traits and different types of juveniles who have committed offenses, namely sexual and non-sexual offenses. Callous-unemotional traits are consistently known to predict aggressive, delinquent, and violent behaviors, but no study so far has examined in a systematic review/meta-analysis the association between CU traits and typologies of individuals who have committed offenses. Considering that the CU traits are more pronounced in juveniles with conduct disorders and that sexual violence is one of the most severe forms of violence, this review intended to analyze whether there are differences in the typologies of individuals who have committed offenses (sexual or non-sexual) in terms of CU traits. More specifically, we reviewed studies that addressed the presence of CU traits in JSOs and/or JGOs, with the main goal of examining which typology have high CU traits.

## 2. Method

This review was not pre-registered. We followed the guidelines and recommendations from the Preferred Reporting Items for Systematic reviews and Meta-Analysis (PRISMA-P [28]).

### 2.1. Inclusion and Exclusion Criteria

Original publications in English, Portuguese, or Spanish that aimed to examine CU traits in male juveniles who have committed offenses during adolescence were included. The samples of the studies should consist of JSOs and/or JGOs, specifically, studies which include JSOs (juveniles who committed sexual offenses) and/or JGOs (juveniles who have engaged in various offending types but not include a sexual offense) and these groups related to a forensic setting (such as description of official records, juvenile detention center, forensic psychiatric facility, sex-offender treatment programs). In accordance with the International Association for the Treatment of Sexual Offenders (IATSO), the age range of JSOs is defined as between 12 and 18 years old [29]. Nevertheless, the age range for this study was defined as 10 to 26 years old (e.g., [30]), because the samples of the articles included young adults in juvenile detention centers, and it was not possible to separate their results from the adolescents. Additionally, only studies, theses, or dissertations that used measures of CU traits in JSOs and/or JGOs, specifically reporting each descriptive category of CU traits, were included.

The defined exclusion criteria were as follows: (a) reviews of the literature, commentaries, case studies, editorials, and qualitative studies; (b) studies with serious methodological issues, i.e., studies not providing sufficient information to allow replication or in which the aims or the methodology implemented were not clear.

### 2.2. Literature Search Procedure

Studies were identified until April 2024 through a search on EBSCOhost, Web of Science, and PubMed databases: Academic Search Complete (*n* = 16) and ERIC (*n* = 2). In order to avoid publication and source selection biases, this search was supplemented by a manual search. The publications were limited to scientific, academic journals, theses, and dissertations published from 1976 to 2023.

The search string used in the systematic search was applied in full text: (“juvenil* sex offend*” OR “adolescen* sex offend*” OR “juvenil* child sex abus*” OR “juvenil* rapist*” OR “juvenil* peer* offend*” OR “juvenil* adult* offend*” OR “adolescent* sexual abus*” OR “p?dophil*” OR “you* sex offend*”) AND (“juvenil* non sex offend*” OR “violent offend*” OR “juvenil* delinquent*” OR “you* offend*”) AND (“callous-unemotional trait*” OR “remors*” OR “poor behavio?r* control*” OR “lack of guilt*” OR “emotion* impair*” OR “affective components of psychopathy” OR “callous lack of empathy” OR “unconcern about performance” OR “shallow affect” OR “shallow emotion*” OR “deficient affect” OR “callousness” OR “uncaring” OR “unemotional”).

### 2.3. Study Selection

A total of 898 studies were identified from all databases and search methods, and 832 were screened after duplicate removal. The abstracts were screened by two independent raters with respect to inclusion and exclusion criteria, resulting in 205 potential studies to be included in the review. Studies disregarded at this stage were either unrelated to the aims of this systematic review or included non-target populations. A total of 627 studies were excluded for the following reasons: publication type (*n* = 416), population type (*n* = 163), and without results of the measures of CU traits (*n* = 48). The inter-rater agreement was calculated, and Cohen’s κ = 0.81 was obtained, indicating an excellent agreement [31]. Disagreements among reviewers were discussed and solved by consensus.

**Figure 1:**
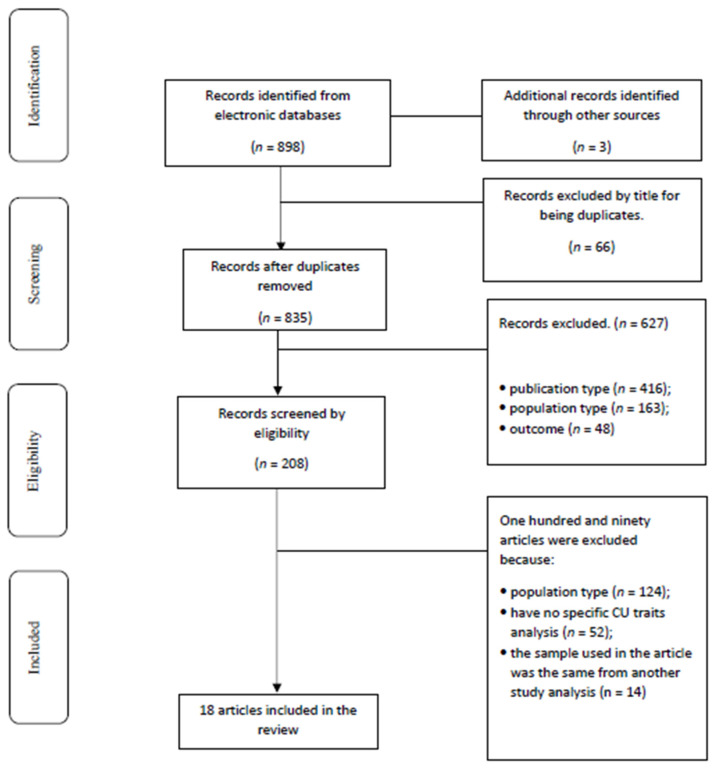
Flowchart of literature review process. In total, 18 papers are cited in the current review.

After analyzing the selected articles in full text, 15 were retained for review, and 190 were excluded for the following reasons: population type (*n* = 124; *n* = 55 refers to samples with juveniles who have engaged in various offending types without differentiation in JSOs or JGOs; *n* = 69 samples without the criteria (e.g., age, other diagnoses, sex)); have no specific CU traits’ analysis (*n* = 52); and the sample used in the article was the same from another study analysis (*n* = 14). In addition, three studies were included from the manual search. In total, this systematic review comprised 18 articles (see Figure 1). The methodological aspects (sample/instruments) and the main conclusions were extracted from each study.

### 2.4. Quality and Risk Bias of Quantitative Studies

The Quantitative Research Assessment Tool was used (QRAT [32]) to assess the methodological quality of the studies included in this review. The QRAT has 12 items, each rated −1, 0, 1, or NA (not applicable) except for the 12th question, where NA is not an option. Thus, the total scores range from −12 to +12. According to the specifications of this tool, studies classified with lower scores should be regarded with more caution, while studies classified with higher scores may reveal a more robust methodology. Most studies included in this review (94.44%) had a score of five or above (see Supplementary Material SI). Almost all studies fully described the variables or concepts of interest, chose variables that are adequate measures of the main concepts, presented quantitative values and appropriate statistical techniques, and addressed alternative explanations. The 18 articles were assessed through QRAT, and only one study had scores below five points.

### 2.5. Exploratory Meta-Analysis

A meta-analysis of studies comparing CU traits in JSOs, JGOs, and/or non-offending juveniles was attempted. However, there were few studies performing comparisons between two different groups (e.g., only six studies compared JSOs to JGOs). Considering that these would not enable the performance of robust analyses, and consequently robust and generalizable results, the meta-analyses were not fully conducted, and the overall effects and other meta-analytical statistics are not reported. To minimize this caveat, pooled means, pooled variances, and pooled standard deviations are still presented in this study, enabling the comparison of the results of future studies with the ones obtained in this review. Note that the pooled mean is a weighted average of multiple means. It is calculated by summing the products of each mean and its corresponding sample size and then dividing this total by the sum of all sample sizes. The pooled variance estimates the overall variance across different populations, even if their means differ. By using the pooled mean and pooled variance, a more accurate estimate of the individual sample variances is obtained. Finally, the pooled standard deviation is found by taking the square root of the pooled variance. This manuscript includes Supplementary Material with information about all studies considered for the meta-analytical procedure (see Supplementary Material SII).

To achieve the objectives of this study, the results of the systematic review are presented below.

## 3. Results

The results section is subdivided into methodological characteristics of the included studies and results regarding CU traits. Although the main goal of this review was to examine which typologies of individuals who have committed offenses (sexual or non-sexual) have high rates of CU traits, results that characterize the multiple types of juveniles who have committed offenses are presented. This information will be important to characterize the different types of juveniles who have committed offenses. Also, with the purpose of easier reading, an acronym list is included in Abbreviations.

### 3.1. Study Designs

All studies used experimental (*n* = 17) or psychometric (*n* = 1) designs to examine the relationships between offenses, sexual, and/or non-sexual, and CU traits in male juveniles. Of the 18 studies analyzed, 16 were cross-sectional, and 2 adopted a longitudinal design. Ten studies presented cross-sectional comparisons between groups with respect to the CU traits.

### 3.2. Studies Characteristics

Details regarding measures used in each study and their main findings are presented in Table 1. The studies included in this systematic review were published from 2006 to 2020, and about 53% of the studies were published in the last decade. Most studies used criminal justice system/juvenile court samples (*n* = 18%), and the participants of all studies were aged between 12 and 26 years old.

As described in Table 1, a variety of psychometric instruments were used to evaluate CU traits. The most frequently used instrument was The Psychopathy Checklist: Youth version (PCL:YV; *n* = 8), followed by the Inventory of Callous-Unemotional Traits (ICU; *n* = 6), the Antisocial Process Screening Device (APSD; *n* = 2), the Youth Psychopathic Traits Inventory (YPI; *n* = 2). In the literature, normative ICU total scores tend to hover around 20 to 26, with scores above 28 to 30 suggesting potentially severe CU and antisocial traits [33]. In this review, average ICU scores of JGOs range between 17.00 and 30.85 and, for JSOs, range between 22.03 and 29.70, while for non-offending juveniles, these scores are 21.25.

The factor structure of the PCL:YV in the included studies is diversified: two-factor model (*n* = 2; [34,35]), and four-factor model (*n* = 5; [36,37,38]). In the case of the PCL:YV [39], four items (Lack of remorse, Callous or lacking empathy, Failure to accept responsibility, Callousness) are pertaining to the affective dimension, and possible scores range from 0 to 8 if it is a single factor (factor 2 on the three or four factor models) or a facet.

With regard to YPI, the average total scores of the Affective scale (assessing Callousness, Unemotional, and Remorselessness) in this review range from 21.88 to 26.40 in JGOs, and in non-offending juveniles, they range from 16.35 to 32.10.

The APSD self-report version has also been used to assess psychopathic traits in adolescents. In the included studies, the average total score of Factor 2 (Callous-unemotional) ranges from 3.91 to 4.34 in JSOs. For JGOs, only one study has presented the APSD score (*M* = 4.97; *SD* = 1.99). Adolescents who score higher on the callous-unemotional factor of the APSD-SR display a greater number and variety of antisocial behaviors and have a greater frequency and variety of violent behaviors.

**Table 1:** Summary of the studies’ characteristics and main findings.

Author, Date/Country	Setting Population	Sample (N)	Controls	Age Range/Mean Age	Typology of Offenses	Outcomes/Measures Used	Main Findings
Barroso et al. [36] Portugal	juvenile detention center	270 JSO = 141 JGO = 129	N/A	12–18 *M* = 14.60; *SD* = 1.60	JSO JGO	PCL:YV	PCL:YV—Factor 2 *: JSO: *M* = 4.11; *SD* = 2.81 JGO: *M* = 4.49; *SD* = 2.61
Boonmann et al. [40] Netherlands	juvenile detention center	487 JSO = 71 JGO = 416	331	JSO *M* = 14.90; *SD* = 1.40 JGO *M* = 14.02; *SD =* 1.0 TC *M* = 15.50; *SD =* 0.80	JSO JGO	YPI	YPI—CU: JSO: *M* = 1.88; *SD* = 0.33 JGO: *M* = 1.76 *SD* = 0.33 N-OJ: *M* = 2.14; *SD* = 0.42
Cale et al. [37] Canada	incarcerated	263 JGO = 223	N/A	12–19 *M* = 16.5; *SD* = 1.60	JGO	PCL:YV	PCL:YV—Factor 2 *: JGO: *M* = 4.40; *SD* = 2.00
Cheng et al. [34] China	juvenile detention center	28 JGO high CU = 15 JGO low CU = 13	17	15–18	JGO	PCL:YV	PCL:YV—Factor 1 * TC: no data JGO high CU: *M* = 11.84; *SD* = 1.05 JGO low CU: *M* = 6.27; *SD* = 1.72
Fanniff and Kimonis [41] USA	juvenile detention center	227 JSO = 108 JGO = 119	N/A	12–19 *M* = 15.73; *SD* = 1.27	JSO JGO	ICU	ICU—U: JSO: *M* = 1.17; *SD* = 0.60 JGO: *M* = 1.60; *SD* = 0.65 ICU—UN: JSO: *M* = 1.52; *SD* = 0.55 JGO: *M* = 1.77; *SD* = 0.58 ICU—C: JSO: *M* = 0.58; *SD* = 0.45 JGO: *M* = 0.81; *SD* = 0.53
Fanniff and Kolko [42] USA	juvenile detention center	JSO-CV = 114 JSO-PV = 50	N/A	*M* = 15.27; *SD* = 1.96	JSO	APSD-SR	APSD—CU JSO-CV: *M* = 3.91; *SD* = 2.2 JSO-PV: *M* = 4.18; *SD* = 2.2
Heynen et al. [43] Germany	juvenile detention center	94	N/A	14–26 *M* = 20.33; *SD* = 2.07	JGO	ICU	ICU—total: *M* = 1.39; *SD* = 0.32
Jusyte et al. [44] Germany	juvenile detention center	23	24	JGO *M* = 19.69; *SD =* 1.05 TC *M* = 19.58; *SD* = 1.50	JGO	YPI ICU	YPI—CU: JGO: *M* = 21.88; *SD* = 7.90 N-OJ: *M* = 16.25; *SD* = 5.61 ICU—total: JGO: *M* = 30.85; *SD* = 11.91 N-OJ: *M* = 21.25; *SD* = 8.02 ICU—U: JGO: *M* = 10.19; *SD* = 5.45 N-OJ: *M* = 7.00; *SD* = 3.59 ICU—UN: JGO: *M* = 9.08; *SD* = 2.81 N-OJ: *M* = 7.75; *SD* = 3.49 ICU—C: JGO: *M* = 11.58; *SD* = 6.48 N-OJ: *M* = 6.50; *SD* = 3.11
Lawing et al. [45] USA	long-term secure custody facility	150	N/A	12–20 *M =* 15.89; *SD* = 1.53	JSO	ICU	ICU—total: *M =* 28.7; *SD* = 7.41
Lindberg et al. [38] Finland	forensic psychiatric examination and Legal Register Center	57	N/A	*M =* 17.60; *SD* = 1.25	JGO	PCL-R	PCL-R Factor 1 *: *M* = 5.50; *SD* = 3.55 PCL-R Facet 2*: *M* = 4.52; *SD* = 2.80
Matlasz et al. [46] USA	juvenile detention center	1216	N/A	13–17 *M* = 15.29; *SD* = 1.29	JGO	ICU	ICU—total: *M* = 26.27; *SD* = 8.03
McCrory et al. [47] UK	forensic assessment and treatment service	237 EO = 75 LO = 132	N/A	12–18	JSO	PCL:YV	PCL:YV—Factor 2 *-EO: *M* = 2.68; *SD* = 2.37 PCL:YV—Factor 2 * -LO: *M* = 2.05; *SD* = 2.13
Morrel and Burton [48] USA	juvenile detention center	191	N/A	13–20 *M* = 17.18; *SD* = 1.71	JSO	ICU	ICU—total: *M* = 22.03; *SD* = 12.28
Parks and Bard [49] USA	juvenile justice agency	156	N/A	12–17 *M* = 14.86; *SD* = 1.24	JSO	PCL:YV	PCL:YV—Factor 2 *^:^ JSO: *M* = 3.31; *SD* = 2.49
Rose et al. [35] USA	juvenile justice systems	JGO = 92 JSO = 21	N/A	13–19 *M* = 17.06; *SD* = NA	JSO JGO	PCL:YV	PCL:YV—Factor 1: JGO: *M* = 7.96; *SD* = 4.25 JSO: *M* = 9.19; *SD* = 3.78
Skilling et al. [50] Canada	juvenile detention center	373 JSO = 78 JGO = 295	N/A	12–20 JSO *M* = 15.08; *SD* = 1.70 JGO *M* = 16.21; *SD* = 1.50	JSO JGO	APSD-SR	APSD—CU: JSO: *M* = 4.34; *SD* = 1.88 JGO: *M* = 4.97; *SD* = 1.99
Yoder et al. [51] USA	juvenile justice systems	JSO = 70 JGO = 130	N/A	13–20 *M* = 17.17; *SD* = 1.81	JSO JGO	ICU	ICU—total JSO: *M* = 0.40; *SD* = 0.43 JGO: *M* = 0.59; *SD* = 0.60
White et al. [52] USA	juvenile detention center	94	N/A	12–18 *M* = 15.22; *SD* = 1.48	JSO	ICU	ICU—total parent-report: *M* = 27.19; *SD* = NA ICU—total self-report: *M =* 21.67; *SD* = NA

Note. APSD = Antisocial Process Screening Device; N-OJ = non-offending juvenile; ICU = Inventory of Callous Unemotional Traits; ICU—U = Inventory of Callous Unemotional Traits, uncaring dimension; ICU—UN = Inventory of Callous Unemotional Traits, unemotional dimension; ICU—C = Inventory of Callous Unemotional, callousness dimension; CU = callous-unemotional; JSO = juvenile who has committed sexual offenses; JGO = juvenile who has committed general offenses; YPI = Youth Psychopathic Traits Inventory; PCL:YV = Hare’s psychopathy checklist—youth version; N/A = not applicable; EO = early onset group; LO = late onset group; APSD-SR = Antisocial Process Screening Device-Self-report; JSO-CV = juvenile who has committed sexual offenses with child victims; JSO-PV = juvenile who has committed sexual offenses with peer/adult victims; * This factor or facet from PCL reflects an affective dimension (callous-unemotional traits) and includes items such as callousness.

### 3.3. CU Traits Assessment in Juveniles Who Have Committed Offenses (Sexual or Non-Sexual)

Eleven studies examined the association between juveniles who have committed offenses (sexual or non-sexual) and CU traits, providing evidence of the association between CU traits and juveniles who have committed at least one offense [34,38,40,41,42,43,44,45,46,47,48].

Through the analysis of the results of each group, JGO samples were the ones who scored higher in instruments that measured CU traits [34,38,43,46]. Specifically, when assessed through PCL:YV, high levels of CU traits were recognized in JGO samples [34,38], as well as severe offense patterns that refer to reactive and instrumental violence (e.g., murder, robbery), followed by reactive violence (e.g., assault, theft) [34]. When CU traits of JGOs were evaluated with the ICU self-report, the average scores ranged between 23.20 and 26.27 [43,46] and were higher than in studies that evaluate JSO samples with this instrument [48,52].

On the contrary, JSO samples usually exhibited low levels of CU traits [40,41,42,43,46]. In this case, studies also reported higher scores on CU scales and/or subscales [45,48]. Lawing et al. [45] reported a high mean score in the ICU self-report (*M* = 28.70; *SD* = 7.41) in a JSO sample that had a history of at least one violent sexual or nonsexual offense and had committed more than one offense (including both sexual and nonsexual offending). Similarly, to the ICU self-report results, White et al. [52] reported a mean of 27.19 (*SD* = not reported) for the total score of the parent-report ICU for JSOs, which was significantly higher than the total score of the self-report ICU (*M* = 21.70; *SD* = not reported). McCrory et al. [47] observed that younger JSOs scored significantly higher on the PCL-YV affective dimension than older JSOs, but their scores were lower than the ones observed in other studies with JGO or JO (sexual and non-sexual offenses) samples. Parks and Bard [49] supported the existence of differences among JSOs with child victims, JSOs with peer/adult victims, and mixed type of offenses on CU traits evaluated with PCL:YV, having identified higher CU levels in the JSO sample with mixed types than the other two groups. On the contrary, a study of Fannif and Kolko [42] found no significant differences between groups of JSOs (i.e., JSOs with child victims in comparison with JSOs with peer/adult victims) on APSD in callous/unemotional dimension tendencies.

### 3.4. Comparisons between Groups

We found eight cross-sectional studies that have made comparisons between different groups assessing CU traits [35,36,40,41,42,44,50,51].

According to Boonmann et al. [40], the JSOs and JGOs scored significantly lower than adolescents from the general population (non-offending juveniles) on YPI unemotional dimension. In general, no differences were observed between JSOs and JGOs regarding the YPI unemotional dimension, but JSOs with other delinquent behaviors have exhibited higher CU traits than JSO solo and JSO child abuses.

Nevertheless, some studies found differences between JGOs and JSOs, with the first scoring higher in CU traits [36,41,50,51], and these traits were associated with a much lower likelihood of having committed a sexual offense [41]. Nonetheless, there was at least one study [35] showing that higher scores in the PCL:YV affective dimension are significantly more common among JSOs than JGOs.

Additionally, when CU traits are evaluated in JGOs compared to non-offending juveniles, it appears that juveniles with conduct problems and more severe behavior patterns have higher CU traits than non-offending juveniles [44].

Although demanding caution due to the heterogeneity of the reviewed studies and to the fact that analyses of significance were not conducted, the results of the pooled means, variances, and standard deviations (see Table 2) seem to corroborate the above-mentioned findings, with the JGOs exhibiting generally higher means of CU traits than JSOs, as measured by the ICU and PCL:YV instruments used. Nevertheless, this meta-analytic approach exhibits that in the YPI total scale, the N-JOs scored higher than the JGOs. Furthermore, the pooled means results show that the JSOs exhibit lower mean scores in measures of CU traits than any other group of juveniles who have committed offenses (also see Supplementary Material SII).

## 4. Discussion

Juvenile delinquency is highly heterogeneous, and it is important to extend the concept of equifinality for understanding antisocial youth by exploring whether additional distinctions can be made within groups who show a childhood onset of their antisocial behavior. Researchers are showing a growing interest in factors associated with the development of aggressive behavior in adolescence, including psychopathic traits, as shown by the recent increase in scientific publications in this area. The affective component of psychopathy, namely callous-unemotional (CU) traits, has been examined in different samples of male adolescents as a risk and/or as a predictive factor for delinquent or antisocial behavior. Still, whether the presence of CU traits constitute a factor capable of distinguishing juveniles who have committed sexual offenses (JSOs) from general offenses is far from being clear. Thus, we conducted a systematic review, following the PRISMA-P guidelines, to gather evidence on the association between CU traits and different types of juveniles who have committed offenses (sexual or non-sexual). Although the performance of statistical analyses was not possible due to the heterogeneity of the studies’ design, the implementation of meta-analytic procedures, pooled means, variances, and standard deviations of CU measures were computed and made available for use as reference, with necessary caution.

It is worth noting the importance of studying the factors and characteristics that may add explanatory power to knowledge about the JSOs and juveniles who have committed general offenses (JGOs). In this line, the specific knowledge of the influence of CU traits on behavior may contribute to clarify the reasons for which some individuals engage in general offenses, while others engage in specific antisocial behavior patterns, such as those related to sexual offenses.

A total of 18 studies examining possible relationships between the CU traits and different types of male juveniles who have committed offenses (sexual or non-sexual), mostly using cross-sectional comparison designs, were reviewed. Evidence of the reviewed studies shows that JGOs present higher indices of CU traits than JSOs [34,36,38,41,42,43,50]. This result is in line with the idea that general delinquency is more likely in those with a grandiose sense of self-worth and a general lack of empathy [13]. One possible explanation could be that sexual crimes involve an emotional and relational component that may not be as present in non-sexual crimes. Individuals who have committed sex offenses may be motivated by specific sexual desires or impulses, which may include distorted needs for intimacy or affection (see [53]). This may imply a certain capacity for emotion, even if in a distorted or pathological way. In addition, individuals who have committed sex offenses may rationalize their actions in ways that still involve a form of empathy, albeit misdirected, while individuals who committed non-sexual offenses may be more rational, cold, and calculating their crimes (e.g., [54]). Finally, individuals who have committed sex offenses may have histories of sexual abuse or early exposure to sexuality, which may not necessarily result in callous-unemotional traits but rather in distortions in sexual perceptions and behaviors [55,56].

Additionally, the majority of studies show that JSOs have lower CU traits compared to other groups of juveniles who have committed offenses [36,41,47,48,49,50,52]. However, some studies show that JSOs have higher levels of CU traits compared to JGOs [35,40].

Only a few of the included studies compared JSOs with JGOs regarding CU traits (e.g., [35,36,40,41,50,51], or assessed these traits within JSOs (e.g., low or high CU traits) [42,45,47,48,49,52], highlighting the need of further studies to strengthen the scientific evidence in this matter. In the 18 studies that were reviewed, only three [40,42,49] compared different types of JSOs (juveniles who have committed sexual offenses individually, juveniles who have committed sexual offenses in groups, and juveniles who have committed child abuse). On the one hand, the results identify differences between these subgroups, with juveniles who have committed sexual offenses in groups showing higher CU traits than both juveniles who have committed sexual offenses individually and juveniles who have committed child abuse [40], and show that JSOs with peer/adults victims and child victims showed high CU trait scores than juveniles with one victim type [49]. This may suggest that sexual offenses committed in groups may be more associated with CU traits, indicating a response by these young people to social rewards. On the other hand, no significant differences between groups of JSOs (i.e., JSOs with child victims in comparison with JSOs with peer/adult victims) were identified [42].

Part of the explanation for the mixed results found in this review could rely on the fact that existing studies defined types of aggression in different ways and used heterogeneous samples. For example, some studies did not provide a detailed description of the sample (i.e., if JSO samples only committed sexual offenses or committed, simultaneously, other offenses) or describe eligible crimes including felony offenses against persons and property, multiple offenses, violent crimes against persons (e.g., murder, rape), property crimes (e.g., arson, burglary), weapons violations, and other crimes [57]. Some studies identified a sexual crime as the presence of higher rates of delinquency in JSOs may have contributed to higher levels of CU traits (e.g., [48]).

Common limitations of systematic reviews also apply to this one. Only studies published in English, Portuguese, and Spanish and identified sources were included, and there is a risk of publication bias. Nonetheless, manual search was performed in order to diminish that risk. The heterogeneity of the methods used in the studies prevents a clearer understanding of the specific influence of CU traits on juveniles’ sexual and other offenses. It is important to note that the quality analysis of all studies shall be considered when interpreting the results. In this case, according to the QRAT [32], the studies that are classified with lower scores may reveal a less robust methodology, but the studies with QRAT scores below five in this systematic review have similar results than studies with more robust methodologies.

Many of the methodological issues of the studies are due to a considerable variety regarding study design (cross-sectional or longitudinal), factor structures for the same psychometric instruments, and methods used in the assessment of CU traits. Most studies use cross-sectional methods to evaluate CU traits, supported by the idea that CU traits are stable throughout life. Nevertheless, this could be a limitation because CU traits are established during development [37]. Generalizations based on the reported findings should be made with caution, considering that different instruments may result in different results regarding the association between CU traits and different types of juveniles who have committed offenses (sexual or non-sexual). As seen above, four different instruments have been identified, and different measures and scoring systems are often reported for the same instrument, making the comparison more costly. Also, the same instruments may present a different factor structure and metric validity in distinct juveniles who have committed offenses, requiring a refinement exercise in the evaluation of the CU traits in different cultures, samples, and ages [58].

Furthermore, most studies have used self-report measures to assess CU traits, which has time-saving advantages. However, the literature has shown that there are disadvantages regarding these types of measures when assessing interpersonal and affective characteristics [59]. As noted by Pechorro et al. [60], youths with high psychopathic traits tend to have a profound lack of self-insight and generally do not see themselves as cold-hearted. In the same line, White et al. [52] found different levels of CU traits for self-report and parent-report regarding the same sample, with parental reports having significantly higher values than self-reports. This reveals a divergence among respondents in the identification of interpersonal and affective characteristics and the tendency of youth with high levels of CU traits to undermine them.

It should also be considered that only a few studies have targeted the specific characteristics of JSOs and their subtypes, focusing on the potential importance of CU traits for sexual offenses in adolescents. Indeed, most studies focus mainly on psychosocial risk factors for antisocial behaviors, with very limited investigation on the relevance of CU traits in the differences between JSOs, JGOs, and groups of non-offending juveniles.

Future studies should consider the combination of different evaluation methods concerning CU traits, namely interviews, reviews of official records, observation, self-report and parent-report or teacher-report questionnaires. Additionally, as mentioned by Seto and Lalumiére [13], studies that distinguish three types of JSOs are scarce: (a) with pedophile sexual interests; (b) rapists; and (c) who have committed child sexual abuses.

Finally, this systematic review showed that most studies do not use non-offending juveniles to compare the data obtained in the evaluations of CU traits with juveniles who have committed offenses. The inclusion of these controls in the methodology may be particularly useful to increase knowledge about CU traits in the community versus JSO and/or JGO samples.

## 5. Conclusions

It is important to identify which variables can explain the onset of sexual offenses and the specific role of such variables in explaining the reasons that lead a teenager to commit sexual crimes, instead of non-sexual offenses or not committing offenses at all. Although, few studies focus on the differences between JSOs, JGOs, and groups of non-offending juveniles in CU traits, the systematic review and the meta-analytic approach suggest that JGOs presents high scores of CU traits, and these traits appear to be related to violent forms of aggression. Nonetheless, its specific role in JSOs seems less relevant. The affective dimensions of psychopathy may be important for differentiating individuals who have committed offenses from other forms of delinquent behavior and, with further study, could provide a target for treatment interventions specifically addressing the mechanisms underlying different types of delinquent behavior.

## Figures and Tables

**Table 2:** Meta-analytic analysis.

Instrument	Statistical Parameter		Groups	
		JSO	JGO	N-OJ
ICU Total				
	Pooled mean	22.35	25.72	---
	Pooled variance	108.29	77.93	---
	Pooled SD	10.41	8.83	---
ICU Uncaring				
	Pooled mean	---	12.38	---
	Pooled variance	---	27.46	---
	Pooled SD	---	5.24	---
ICU Unemotional				
	Pooled mean	---	7.84	---
	Pooled variance	---	7.61	---
	Pooled SD	---	2.76	---
ICU Callousness				
	Pooled mean	---	7.98	---
	Pooled variance	---	25.78	---
	Pooled SD	---	5.08	---
YPI CU				
	Pooled mean	---	26.16	31.03
	Pooled variance	---	26.41	39.15
	Pooled SD	---	5.14	6.26
APSD-SR				
	Pooled mean	4.10	---	---
	Pooled variance	4.42	---	---
	Pooled SD	2.10	---	---
PCL:YV—Factor 2				
	Pooled mean	3.11	4.43	---
	Pooled variance	6.15	5.42	---
	Pooled SD	2.48	2.33	---
PCL:YV—Factor 1				
	Pooled mean	---	8.17	---
	Pooled variance	---	14.52	---
	Pooled SD	---	3.81	---

Note. JSO = juvenile who has committed sexual offenses; JGO = juvenile who has committed general offenses; N-OJ = non-offending juvenile; SD = Standard deviation; ICU = Inventory of Callous-Unemotional Traits; PCL:YV = Hare’s psychopathy checklist—youth version; APSD = Antisocial Process Screening Device. YPI = Youth Psychopathic Traits Inventory Callous-unemotional; Characteristics of used instruments: ICU Total = 24 items, Likert scale 0–3; ICU Uncaring = 8 items, Likert scale 0–3; Likert scale 1–4; ICU Unemotional = 5 items, Likert scale 0–3; ICU Callousness = 9 items, Likert scale 0–3; PCL:YV—Factor 2 = 4 items, Likert scale 0–2; PCL:YV—Factor 1 = 8 items, Likert scale 0–2; APSD-SR = 6 items, Likert scale 0–2; YPI CU = 15 items, Likert scale 1–4. The studies considered in each analysis are found in the Supplementary Material SII.

## Supplementary Materials

The following supporting information can be downloaded at: https://www.mdpi.com/article/10.3390/bs14070525/s1.

## Abbreviations

APSD	Antisocial Process Screening Device
CU	Callous-Unemotional
IATSO	International Association for the Treatment of Sexual Offenders
ICU	Inventory of Callous-Unemotional Traits
JGO	Juveniles who have committed General Offenses
JSO	Juveniles who have committed Sexual Offenses
N-OJ	non-offending juvenile
PCL:YV	The Psychopathy Checklist: Youth version
PRISMA-P	Preferred Reporting Items for Systematic reviews and Meta-Analysis
QRAT	Quantitative Research Assessment Tool
YPI	Youth Psychopathic Traits Inventory

## References

[1] Broidy L., Nagin D., Tremblay R., Bates J., Brame B., Dodge K. (2003). Developmental Trajectories of Childhood Disruptive Behaviors and Adolescent Delinquency: A Six-Site, Cross-National Study. Dev. Psychol..

[2] Muñoz L., Frick P. (2012). Callous-Unemotional Traits and Their Implication for Understanding and Treating Aggressive and Violent Youths. Crim. Justice Behav..

[3] Manita C., Ribeiro C., Peixoto C. (2009). Violência Doméstica: Compreender Para Intervir: Guia de Boas Práticas Para Profissionais Das Forças de Segurança.

[4] Kuo C., Mathews C., Abrahams N. (2018). Sexual Violence as a Global Health Problem: Current Evidence and Future Directions. Sexual Assault Risk Reduction and Resistance.

[5] Rich P., Rich P. (2003). The juvenile sexual offender: Commonalities and characteristics. Juvenile Sexual Offenders: Understanding, Assessing and Rehabilitating.

[6] Worling J. (2012). The assessment and treatment of deviant sexual arousal with adolescents who have offended sexually. J. Sex. Aggress..

[7] Aebi M., Giger J., Plattner B., Metzke C.W., Steinhausen H.C. (2013). Problem coping skills, psychosocial adversities and mental health problems in children and adolescents as predictors of criminal outcomes in young adulthood. Eur. Child Adolesc. Psychiatry.

[8] Malin H., Saleh F., Grudzinskas A. (2014). Recent research related to juvenile sex offending: Findings and directions for further research. Curr. Psychiatry Rep..

[9] DeLisi M., Piquero A.R. (2011). New frontiers in criminal careers research, 2000–2011: A state-of-the-art review. J. Crim. Justice.

[10] Frick P., White S. (2008). Research review: The importance of callous unemotional traits for developmental models of aggressive and antisocial behavior. J. Child Psychol. Psychiatry.

[11] Johanson M., Vaurio O., Tiihonen J., Lähteenvuo M. (2020). A systematic literature review of neuroimaging of psychopathic traits. Front. Psychiatry.

[12] Frick P., Ray J., Thornton L., Kahn R. (2014). Can callous-unemotional traits enhance the understanding, diagnosis, and treatment of serious conduct problems in children and adolescents? A comprehensive review. Psychol. Bull..

[13] Seto M., Lalumière M. (2010). What is so special about male adolescent sexual offending? A review and test of explanations through meta-analysis. Psychol. Bull..

[14] Calkins S.D., Keane S. (2009). Developmental origins of early antisocial behavior. Dev. Psychopathol..

[15] Frick P. (2012). Developmental pathways to conduct disorder: Implications for future directions in research, assessment, and treatment. J. Clin. Child Adolesc. Psychol..

[16] Loney B., Frick P., Clements C., Ellis M., Kerlin K. (2003). Callous-unemotional traits, impulsivity, and emotional processing in adolescents with antisocial behavior problems. J. Clin. Child Adolesc. Psychol..

[17] Neo B., Kimonis E. (2021). Callous–unemotional traits linked to earlier onset of self-reported and official delinquency in incarcerated boys. Law Hum. Behav..

[18] Thornton L., Frick P., Ray J., Wall Myers T., Steinberg L., Cauffman E. (2019). Risky sex, drugs, sensation seeking, and callous unemotional traits in justice-involved male adolescents. J. Clin. Child Adolesc. Psychol..

[19] Ray J., Thornton L., Frick P., Steinberg L., Cauffman E. (2016). Impulse control and callous-unemotional traits distinguish patterns of delinquency and substance use in justice involved adolescents: Examining the moderating role of neighborhood context. J. Abnorm. Child Psychol..

[20] Knight R., Guay J., Patrick C.J. (2006). The role of psychopathy in sexual coercion against women. Handbook of Psychopathy.

[21] Essau C., Sasagawa S., Frick P. (2006). Callous-unemotional traits in a community sample of adolescents. Assessment.

[22] Mann F., Tackett J., Tucker-Drob E., Harden K. (2017). Callous-Unemotionall traits moderate genetic and environmental influences in rule-breaking and aggression: Evidence for gene x trait interaction. Clin. Psychol. Sci..

[23] Caputo A., Frick P., Brodsky S. (1999). Family violence and juvenile sex offending: The potential mediating role of psychopathic traits and negative attitudes toward women. Crim. Justice Behav..

[24] Baskin-Sommers A., Curtin J., Newman J. (2015). Altering the cognitive-affective dysfunctions of psychopathic and externalizing offender subtypes with cognitive remediation. Clin. Psychol. Sci. A J. Assoc. Psychol. Sci..

[25] Edens J., Campbell J., Weir J. (2007). Youth psychopathy and criminal recidivism: A meta-analysis of the Psychopathy Checklist measures. Law Hum. Behav..

[26] Frick P., Dickens C. (2006). Current perspectives on conduct disorder. Curr. Psychiatry Rep..

[27] Frick P., Viding E. (2009). Antisocial behavior from a developmental psychopathology perspective. Dev. Psychopathol..

[28] Shamseer L., Moher D., Clarke M., Ghersi D., Liberati A., Petticrew M., Shekelle P., Stewart L., the PRISMA-P Group (2015). Preferred reporting items for systematic review and meta-analysis protocols (PRISMA-P) 2015: Elaboration and explanation. Br. Med. J..

[29] Miner M., Borduin C., Prescott D., Bovensmann H., Schepker R., Du Bois R., Schladale J., Eher R., Schmeck K., Langfedt T. (2006). Standards of care for juvenile sexual offenders of the International Association for the Treatment of Sexual Offenders. Sex. Offender Treat..

[30] Andershed H., Köhler D., Louden J., Hinrichs G. (2008). Does the three-factor model of psychopathy identify a problematic subgroup of young offenders?. Int. J. Law Psychiatry.

[31] Landis R., Koch G. (1977). The measurement of observer agreement for categorical data. Biometrics.

[32] Child Care & Early Education Research Connections Quantitative Research Assessment Tool 2019. https://www.researchconnections.org/childcare/datamethods/downloads/quantitativeresearch.pdf.

[33] Aghajani M., Klapwijk E., van der Wee N., Veer I., Rombouts S., Boon A., Beelen P., Pompa A., Vermeiren R., Colins O. (2016). Disorganized amygdala networks in conduct-disordered juvenile offenders with callous-unemotional traits. Biol. Psychiatry.

[34] Cheng Y., Hung A., Decety J. (2012). Dissociation between affective sharing and emotion understanding in juvenile psychopaths. Dev. Psychopathol..

[35] Rose K., Woodworth M., Minton J. (2020). An exploration of individual differences in a sample of youth charged with violent sexual and non-sexual crimes. Psychiatry Psychol. Law.

[36] Barroso R., Figueiredo P., Ramião E., Fanti K. (2020). Using psychopathy to identify differences between variants of juveniles who have committed sexual offenses. J. Sex. Aggress..

[37] Cale J., Lussier P., McCuish E., Corrado R. (2015). The prevalence of psychopathic personality disturbances among incarcerated youth: Comparing serious, chronic, violent and sex offenders. J. Crim. Justice.

[38] Lindberg N., Laajasalo T., Holi M., Putkonen H., Weizmann-Henelius G., Häkkänen-Nyholm H. (2009). Psychopathic traits and offender characteristics—A nationwide consecutive sample of homicidal male adolescents. BMC Psychiatry.

[39] Forth A.E., Kosson D., Hare R. (2003). The Hare Psychopathy Checklist: Youth Version.

[40] Boonmann C., Jansen L., Hart-Kerkhoffs L., Vahl P., Hillege S., Doreleijers T., Vermeiren R. (2015). Self-Reported psychopathic traits in sexually offending juveniles compared with generally offending juveniles and general population youth. Int. J. Offender Ther. Comp. Criminol..

[41] Fanniff A., Kimonis E. (2014). Juveniles who have committed sexual offenses: A special group?. Behav. Sci. Law.

[42] Fanniff A.M., Kolko D.J. (2011). Victim age-based subtypes of juveniles adjudicated for sexual offenses. Sex. Abus. A J. Res. Treat..

[43] Heynen E., Van der Helm G., Stams G., Korebrits A. (2016). Measuring empathy in a German youth prison: A validation of the German version of the basic empathy scale (bes) in a sample of incarcerated juvenile offenders. J. Forensic Psychol. Pract..

[44] Jusyte A., Mayer S., Künzel E., Hautzinger M., Schönenberg M. (2015). Unemotional traits predict early processing deficit for fearful expressions in young violent offenders: An investigation using continuous flash suppression. Psychol. Med..

[45] Lawing K., Frick P., Cruise K. (2010). Differences in offending patterns between adolescent sex offenders high or low in callous–unemotional traits. Psychol. Assess..

[46] Matlasz T.M., Frick P.J., Robertson E.L., Ray J.V., Thornton L.C., Wall Myers T.D., Steinberg L., Cauffman E. (2020). Does self-report of aggression after first arrest predict future offending and do the forms and functions of aggression matter?. Psychol. Assess..

[47] McCrory E., Hickey N., Farmer E., Vizard E. (2008). Early-onset sexually harmful behaviour in childhood: A marker for life-course persistent antisocial behaviour?. J. Forensic Psychiatry Psychol..

[48] Morrell L.M., Burton D.L. (2014). An exploration of psychopathy in self-report measures among juvenile sex offenders. Int. J. Offender Ther. Comp. Criminol..

[49] Parks G.A., Bard D.E. (2006). Risk Factors for Adolescent Sex Offender Recidivism: Evaluation of Predictive Factors and Comparison of Three Groups Based Upon Victim Type. Sex. Abus. A J. Res. Treat..

[50] Skilling T.A., Doiron J.M., Seto M.C. (2011). Exploring differences in youth and parent reports of antisociality among adolescent sexual and nonsexual offenders. Psychol. Assess..

[51] Yoder J., Grady M.D., Brown A., Dillard R. (2019). Criminogenic needs as intervening factors in the relation between insecure attachments and youth sexual violence. Sex. Abus..

[52] White S.F., Cruise K.R., Frick P.J. (2009). Differential correlates to self-report and parent-report of callous-unemotional traits in a sample of juvenile sexual offenders. Behav. Sci. Law.

[53] Marshall W.L., Barbaree H.E., Marshall W.L., Laws D.R., Barbaree H.E. (1990). An integrated theory of the etiology of sexual offending. Handbook of Sexual Assault: Issues, Theories, and Treatment of the Offender.

[54] Samenow S. (2014). Inside The Criminal Mind.

[55] D’Urso G., Petruccelli I., Costantino V., Zappulla C., Pace U. (2018). The role of moral disengagement and cognitive distortions toward children among sex offenders. Psychiatry Psychol. Law.

[56] Ward T. (2000). Sexual offenders’ cognitive distortions as implicit theories. Aggress. Violent Behav..

[57] Cauffman E., Kimonis E., Dmitrieva J., Monahan K. (2009). A multimethod assessment of juvenile psychopathy: Comparing the predictive utility of the PCL:YV, YPI, and NEO PRI. Psychol. Assess..

[58] Hillege S., de Ruiter C., Smits N., van der Baan H., Das J. (2011). Structural and Metric Validity of the Dutch Translation of Psychopathy Checklist: Youth Version (PCL:YV). Int. J. Forensic Ment. Health.

[59] Lee Z., Vincent G.M., Hart S.D., Corrado R.R. (2003). The Validity of the Antisocial Process Screening Device as a Self-report Measure of Psychopathy in Adolescent Offenders. Behav. Sci. Law.

[60] Pechorro P., Ribeiro da Silva D., Andershed H., Rijo D., Abrunhosa R.G. (2016). The Youth Psychopathic Traits Inventory: Measurement Invariance and Psychometric Properties among Portuguese Youths. Int. J. Environ. Res. Public Health.

