# Supplementary material for: Do Juveniles Who Have Committed Sexual Offenses Have Higher Callous-Unemotional Traits Compared to Juveniles Who Have Committed General Offenses? A Systematic Review
Source: Behav Sci (Basel). 2024 Jun 24;14(7):525. doi: 10.3390/bs14070525 (PMC11273882; doi:10.3390/bs14070525)
Supplement: Supplementary file 1 [file behavsci-14-00525-s001.zip › Supplementary material SII_.pdf]

Supplementary material SII

Results of the meta-analytic procedure

Groups/Instruments	Authors	Country	N	Mean	SD	Variance	Pooled Mean	Pooled Variance	Pooled SD
**JSO**									
ICU total							22.35	108.29	10.41
	Lawing et al. [45]	United States	150	28.70	7.41	54.91			
	Morrel & Burton [48]	United States	191	22.03	12.28	150.80			
	Yoder et al. [51]	United States	70	9.60	10.32	106.50			
PCL:YV Factor 2							3.11	6.15	2.48
	Barroso et al. [36]	Portugal	141	4.11	2.81	7.89			
	McCrory et al. [47]	UK	75	2.68	2.37	5.62			
	McCrory et al. [47]	UK	132	2.05	2.13	4.54			
	Parks and Bard [49]	United States	156	3.31	2.49	6.20			
APSD							4.10	4.42	2.10
	Fanniff & Kolko [42]	United States	114	3.91	2.20	4.84			
	Fanniff & Kolko [42]	United States	50	4.18	2.20	4.84			
	Skilling et al. [50]	Canada	78	4.34	1.88	3.53			
**JGO**									
ICU Total							25.72	77.93	8.83
	Heynen et al. [43]	Germany	94	33.36	7.68	58.98			
	Jusyte et al. [44]	Germany	23	30.85	11.91	141.85			
	Matlasz et al. [46]	United States	1216	26.27	8.03	64.48			

		Yoder et al. [51]	United States	130	14.16	14.40	207.36			
ICU Uncaring								12.38	27.46	5.24
		Fanniff & Kimonis [41]	United States	119	12.80	5.20	27.04			
		Jusyte et al. [44]	Germany	23	10.19	5.45	29.70			
ICU Unemotional								7.84	7.61	2.76
		Fanniff & Kimonis [41]	United States	119	7.60	2.75	7.56			
		Jusyte et al. [44]	Germany	23	9.08	2.81	7.90			
ICU Callousness								7.98	25.78	5.08
		Fanniff & Kimonis [41]	United States	119	7.29	4.77	22.75			
		Jusyte et al. [44]	Germany	23	11.58	6.48	41.99			
PCL:YV Factor 2								4.43	5.42	2.33
		Barroso et al. [36]	Portugal	129	4.49	2.61	7.89			
		Cale et al. [37]	Canada	223	4.40	2.00	4.00			
PCL:YV Factor 1								8.17	14.52	3.81
		Cheng et al. [34]	China	15	6.27	1.72	2.96			
		Cheng et al. [34]	China	13	11.84	1.05	1.10			
		Rose et al. [35]	United States	92	7.96	4.25	18.06			
YPI								26.16	26.41	5.14
		Boonmann et al. [40]	Netherlands	416	26.40	4.95	24.50			
		Jusyte et al. [44]	Germany	23	21.88	7.90	62.41			
	**N-OJ**									
YPI								31.03	39.15	6.26
		Boonmann et al. [40]	Netherlands	331	32.10	6.30	39.69			
		Jusyte et al. [44]	Germany	24	16.25	5.61	31.47			

Note. JSO = juvenile who have committed sexual offenses; JGO = juvenile who have committed general offenses; JO = juvenile who have committed either sexual or non-sexual offenses; N-OJ = non-offending juvenile; ICU = Inventory of Callous-Unemotional Traits; ICU Total[1] = 24 items, Likert scale 1–4; PCL:YV = Hare's psychopathy checklist – youth version; PCL:YV Factor 1 = 8 items, Likert scale 0-2; APSD = Antisocial Process Screening Device. YPI = Youth Psychopathic Traits Inventory; Characteristics of used instruments: ICU Total = 24 items, Likert scale 0–3; ICU Uncaring = 8 items, Likert scale 0–3; Likert scale 1-4; ICU Unemotional = 5 items, Likert scale 0–3; ICU Callousness = 9 items, Likert scale 0–3; PCL:YV Factor 2 = 4 items, Likert scale 0–2; APSD-SR = 6 items, Likert scale 0–2; YPI CU = 15 items, Likert scale 1–4.
