# Supplementary material for: Do Juveniles Who Have Committed Sexual Offenses Have Higher Callous-Unemotional Traits Compared to Juveniles Who Have Committed General Offenses? A Systematic Review
Source: Behav Sci (Basel). 2024 Jun 24;14(7):525. doi: 10.3390/bs14070525 (PMC11273882; doi:10.3390/bs14070525)
Supplement: Supplementary file 1 [file behavsci-14-00525-s001.zip › Supplementary material SI_.pdf]

Supplementary material SI

Quality and risk bias of quantitative studies

Nr.	Study ID	**Item 1**	**Item 2**	**Item 3**	**Item 4**	**Item 5**	**Item 6**	**Item 7**	**Item 8**	**Item 9**	**Item 10**	**Item 11**	**Item 12**	**Total score**
1	Barroso et al. (2020)	1	0	0	-1	1	1	1	-1	1	1	1	1	6
2	Boonmann et al (2015)	1	0	1	-1	1	1	1	0	1	1	0	1	7
3	Cale et al. (2015)	1	0	0	-1	1	1	1	0	1	1	1	-1	5
4	Cheng et al. (2012)	0	0	-1	-1	1	1	1	1	1	1	1	1	6
5	Fanniff & Kimonis (2014)	1	0	0	-1	1	1	1	0	1	1	1	1	7
6	Fanniff & Kolko (2012)	1	0	0	-1	1	1	1	0	1	1	1	-1	5
7	Heynen et al. (2016)	0	0	-1	1	1	1	1	0	1	1	1	1	7
8	Jusyte et al. (2015)	0	0	-1	1	1	1	1	1	1	1	1	1	8
9	Lawing et al. (2010)	1	0	0	-1	1	1	1	0	1	1	1	1	7
10	Lindberg et al. (2009)	0	1	-1	-1	1	1	1	0	1	1	1	1	6
11	Matlasz et al. (2020)	1	0	1	1	1	1	1	1	1	1	1	1	11

12	McCrory et al. (2008)	1	0	0	1	1	1	1	0	1	1	1	-1	7
13	Morrel & Burton (2014)	1	0	0	1	1	1	1	0	-1	1	1	-1	5
14	Parks and Bard (2006)	1	0	0	-1	1	1	1	0	1	1	1	-1	5
15	Rose et al. (2020)	0	0	-1	1	1	1	1	-1	1	1	1	-1	4
16	Skilling et al. (2011)	1	0	0	1	1	1	1	0	1	1	1	1	9
17	Yoder et al. (2020)	0	0	0	1	1	1	1	1	1	1	1	1	9
18	White et al. (2009)	0	0	-1	1	1	1	-1	0	1	1	1	1	5

Legend: Item 1 – Population; Item 2 - Sample size; Item 3 - Randomized Selection of Participants; Item 4 - Response and Attrition Rate; Item 5 - Main Variables or Concepts; Item 6 - Operationalization of Concepts; Item 7 -Numeric Tables; Item 8 – Missing Data; Item 9 – Appropriateness of Statistical Techniques; Item 10 – Omitted Variable Bias; Item 11 – Analysis of main Effect Variables; Item 12 – Ethical Approval.
